# Significant association between tumor mutational burden and immune-related adverse events during immune checkpoint inhibition therapies

**DOI:** 10.1007/s00262-020-02543-6

**Published:** 2020-03-09

**Authors:** Csaba Kerepesi, Tibor Bakacs, Ralph W. Moss, Shimon Slavin, Colin C. Anderson

**Affiliations:** 1 Institute for Computer Science and Control (SZTAKI), Kende u 13-17, Budapest, 1111 Hungary; 2 PRET Therapeutics Ltd., Budapest, 1124 Hungary; 3 Moss Reports, 104 Main Street Unit 1422, Blue Hill, ME 04614-1422 USA; 4 Biotherapy International, The Center for Innovative Cancer Immunotherapy and Cellular Medicine, Weizmann Center, 14 Weizmann Street Floor 15, Suite 1503, 64239 Tel Aviv, Israel; 5 Departments of Surgery and Medical Microbiology and Immunology, Alberta Diabetes Institute, Alberta Transplant Institute, University of Alberta, Edmonton, AB T6G 2E1 Canada; 6 Present Address: Brigham and Women’s Hospital, Harvard Medical School, 77 Avenue Louis Pasteur, Boston, MA 02115 USA

**Keywords:** Immune-related adverse event, Immune checkpoint inhibition, Tumor mutation burden, Graft-versus-host disease, FAERS

## Abstract

More than 2000 immuno-oncology agents are being tested or are in use as a result of the cancer immunotherapy revolution. Manipulation of co-inhibitory receptors has achieved tumor eradication in a minority of patients, but widespread immune-related adverse events (irAEs) compromised tolerance to healthy self-tissues in the majority. We have proposed that a major mechanism of irAEs is similar to a graft-versus-malignancy effect of graft-versus-host disease. To verify our hypothesis, we retrieved post-marketing data of adverse events from the U.S. Food and Drug Administration Adverse Event Reporting System. A significant positive correlation was revealed in 7677 patients between the reporting odds ratio of irAEs during immune checkpoint inhibitor therapy and the corresponding tumor mutational burden across 19 cancer types. These results can be interpreted to mean that the ICI drugs unleashed T cells against “altered-self,” self, and tumors resulting in better overall survival.

**Electronic supplementary material:**

The online version of this article (10.1007/s00262-020-02543-6) contains supplementary material, which is available to authorized users.

Bomze et al. found a significant association between tumor mutational burden (TMB) and immune-related adverse events (irAEs) across different cancer types during nivolumab or pembrolizumab anti-programmed cell death-1 (anti-PD-1) therapy [1]. This finding seems to support our hypothesis that the widespread irAEs are primarily due to a mechanism similar to autologous-graft-versus-host-like-disease (auto-GVHD), part of which is a graft-versus-malignancy (GVM) effect responsible for induction of anti-cancer effects that can result in tumor eradication [2]. Since different agents targeting PD-1, PD-L1, or cytotoxic T-lymphocyte-associated protein 4 (CTLA-4) may involve distinct mechanisms, we examined whether the correlation of TMB and irAEs with nivolumab or pembrolizumab therapy is applicable to immune checkpoint blockade more generally. Formal proof that irAEs are associated with a significantly longer survival is available for anti-PD-1 (pembrolizumab) [3]. Notwithstanding, the highest efficacy can only be achieved with *concurrent* ipilimumab and nivolumab blockade, which is also inseparable from the highest rate of irAEs. The record 3-year overall survival (OS) rate of 63% has been observed in patients with metastatic melanoma who were treated with combined ipilimumab and nivolumab blockade [4]. For this spectacular result, however, a heavy price had to be paid: tolerance to healthy self-tissues was severely compromised. Treatment-related irAEs, of any-grade, were reported in 92.6% of patients, 40.4% of which were grade 3 and 4, leading to discontinuation in 24.5% of patients and one death. Not unexpectedly, a meta-analysis including 48 trials with 7936 patients who were treated with nivolumab or nivolumab plus ipilimumab raised the question whether the deleterious effects of severe irAEs outweigh the benefit from the addition of ipilimumab [5]. We have therefore also included in our analysis the anti-CTLA-4/anti-PD-1 combination therapy.

We strictly followed Bomze et al. [1] and retrieved post-marketing data of adverse events from the U.S. Food and Drug Administration Adverse Event Reporting System (FAERS) from July 1, 2014, to March 31, 2019. We considered cancers only for which there were at least 100 cases of adverse events during immune checkpoint inhibitor therapy reported in FAERS. To assess the risk of a patient developing any irAE (Supplementary Table S1) as defined by the reporting odds ratios (RORs) [6] we compared the odds of reporting these irAEs in patients treated either with anti-CTLA-4 (ipilimumab, tremelimumab), anti-PD-1 (nivolumab, pembrolizumab, cemiplimab, pidilizumab, spartalizumab, tislelizumab, toripalimab), anti-PD-L1 (avelumab, durvalumab, atezolizumab), or combination therapy with the odds for all other drugs in the database. The median number of coding somatic mutations per megabase in tumor tissue (referred as tumor mutation burden, TMB) for each cancer type was obtained from previously published comprehensive genomic profiling [7, 8]. GVHD symptoms and FAERS search terms were determined according to Jagasia et al. [9] and collected into Supplementary Table S2.

Our search strategy identified a total of 80,193 adverse events (AEs) of all types in 28,092 patients reported as treated with immune checkpoint inhibitor therapy for 19 different cancer types. Of these patients, 7677 had at least 1 irAE (proportion: 27.3%; proportion per cancer: 9.1–36%). The comparator group comprised 17,069,184 AE reports from 5,937,270 patients. Of these patients, 428,922 had at least 1 irAE (proportion: 7.2%; odds: 0.078). Our analysis revealed a significant positive correlation between the ROR of reporting an irAE during immune checkpoint inhibitor therapy and the corresponding TMB across multiple cancer types, with a higher ROR of irAE associated with a higher median number of coding somatic mutations per megabase of DNA (Fig. 1; Pearson correlation coefficient *r* = 0.68, *p* = 0.0012). Importantly, significant positive association was also demonstrated when exclusively all anti-PD-1/anti-CTLA-4 combination treatments were included (Fig. 2; *r* = 0.661, *p* = 0.0127). Since there is an extensive overlap between the irAEs of ICIs [10] and the symptoms of chronic graft-versus-host disease (GVHD) [9], we investigated the correlation between the chronic GVHD-related AE reporting odds ratio (ROR) and the corresponding TMB across multiple cancer types. A significant positive association was demonstrated (Fig. 3; *r* = 0.466, *p* = 0.0217). This association appears not to be widely recognized. We found 3172 and 4102 papers with the keywords either <ipilimumab> or <nivolumab>, surprisingly, only 13 and 17 papers using the keywords either <ipilimumab and GVHD> or <nivolumab and GVHD>, respectively (PubMed search as of November 2019). While out of these 30 papers 27 were concerned with the effect of ICI drugs on GVHD after allogeneic hematopoietic stem cell transplantation, only our own papers proposed the possibility of an ICI drug induced auto-GVHD reaction [2, 11, 12].

**Fig. 1:**
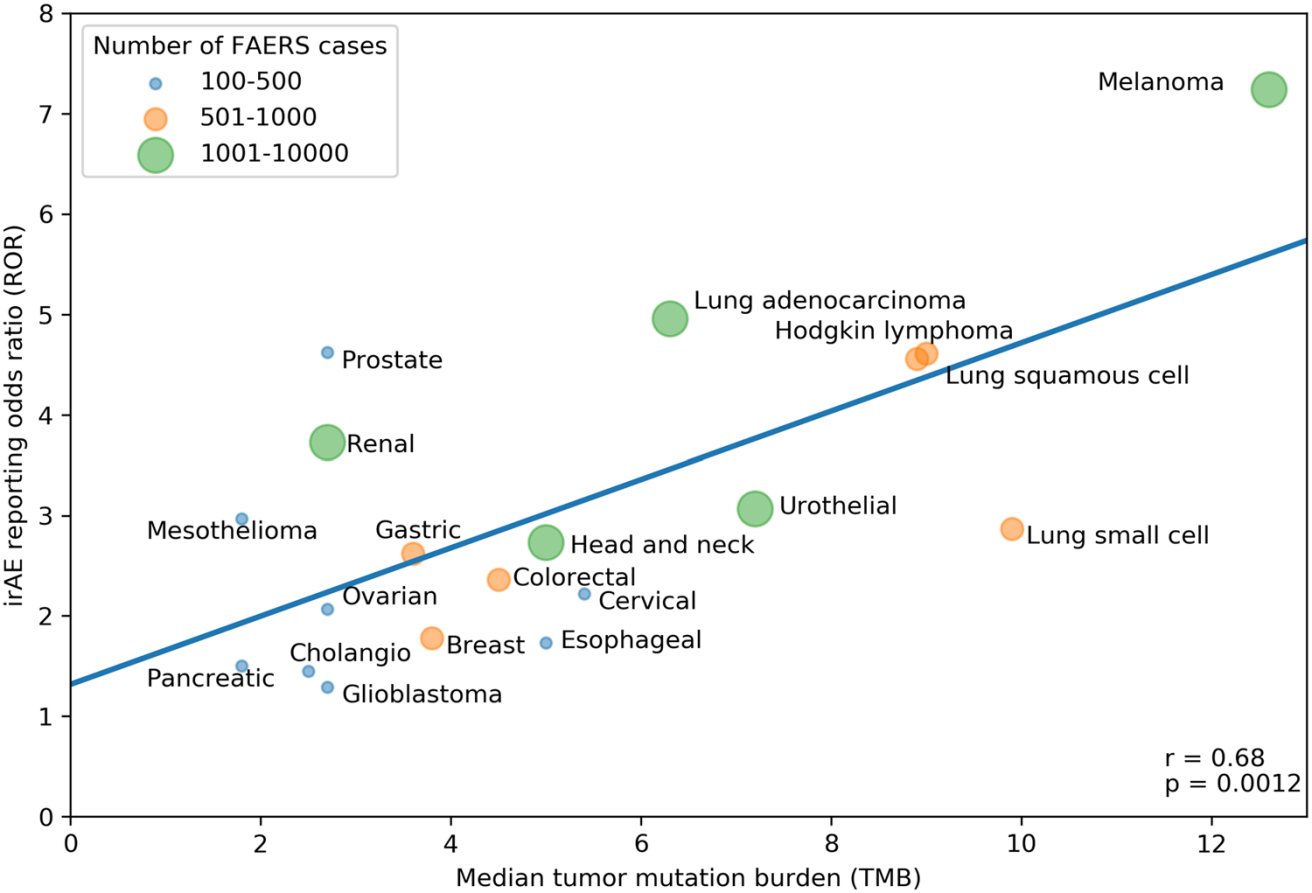
Association between tumor mutational burden (median number of coding somatic mutations per megabase) and immune-related adverse events during either Anti-CTLA-4, Anti-PD-1, Anti-PD-L1 or combination therapy. The straight line represents the linear fit. Circle size and color represent the total number of FAERS cases for each cancer type. Pearson correlation coefficient (*r*) and the corresponding *p* value are shown at the bottom-right of the figure. The *p* value means the probability of getting higher *r* with random ROR values

**Fig. 2:**
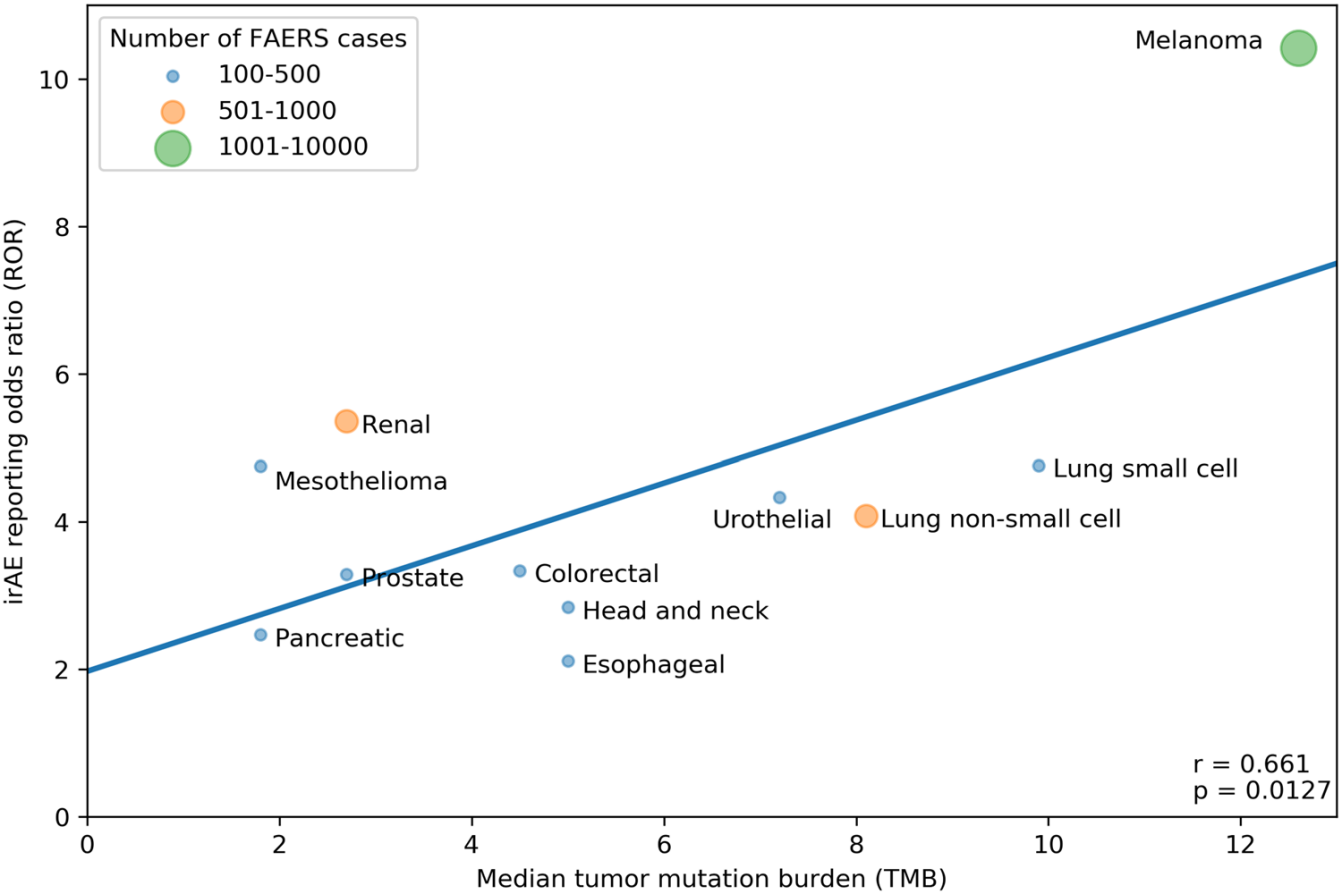
Association between tumor mutational burden (median number of coding somatic mutations per megabase) and immune-related adverse events during Anti-CTLA-4/Anti-PD-1 combination therapy. The straight line represents the linear fit. Circle size and color represent the total number of FAERS cases for each cancer type. Pearson correlation coefficient (*r*) and the corresponding *p* value are shown at bottom-right of the figure. The *p* value means the probability of getting higher *r* with random ROR values

**Fig. 3:**
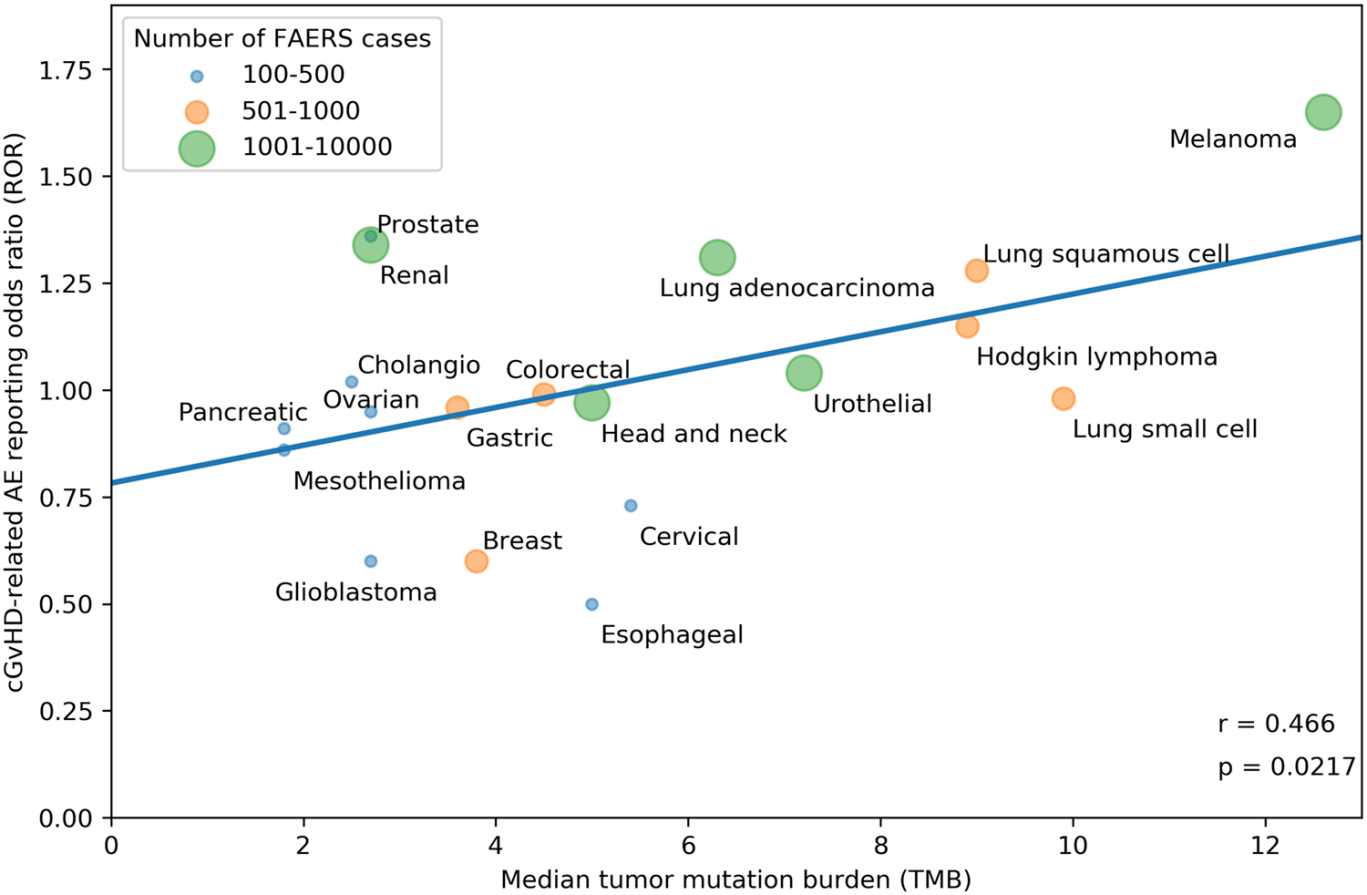
Association between tumor mutational burden (median number of coding somatic mutations per megabase) and chronic graft-versus-host disease (cGVHD) related adverse events (AEs) during either Anti-CTLA-4, Anti-PD-1, Anti-PD-L1 or combination therapy. The straight line represents the linear fit. Circle size and color represent the total number of FAERS cases for each cancer type. Pearson correlation coefficient (*r*) and the corresponding *p* value are shown at the bottom-right of the figure. The *p* value means the probability of getting higher *r* with random ROR values

High TMB, representing genomic instability, has the potential to induce neoantigen production. Alexandrov et al. [13] analyzed 4,938,362 mutations from 7042 cancers and extracted more than 20 distinct mutational signatures. The prevalence of somatic mutations was found to be highest in melanoma, lung squamous carcinoma, lung adenocarcinoma, bladder carcinoma, lung small cell carcinoma, esophageal carcinoma, colorectal carcinoma, cervix carcinoma, head and neck carcinoma. Melanomas have the highest mutational burdens (up to 100 mutations per megabase) as compared with other solid tumors.

The Kaplan–Meier curves for overall survival demonstrated a significant clinical benefit from CTLA-4 blockade in patients with melanoma with a neoepitope (nonsynonymous coding mutations) signature over those without the signature [14]. Consistent with this, Wang et al. [15], demonstrated in patients with advanced non-small cell lung carcinoma (NSCLC) that high TMB, estimated by circulating tumor DNA in blood (bTMB), was associated with superior progression-free survival and objective response rates to anti-PD-1 and anti-PD-L1 therapy compared to patients with low bTMB. Samstein et al. [16] also found that checkpoint inhibitors were more likely to halt tumor growth in patients with high TMB than in those with fewer mutations.

Our analysis indicates that cancers with a high TMB, such as melanoma, small cell and non-small cell lung cancers are associated with a higher irAE ROR during immune checkpoint inhibitor therapy. Since in our view neither anti-CTLA-4 nor anti-PD-1 antibodies are tumor specific [17], the development of irAEs depends upon the derangement of self-tolerance. Samstein et al. hypothesized that higher mutation load is associated with a higher number of tumor neoantigens that facilitate immune recognition and the development of an antitumor immune response [16]. In contrast, we proposed that this situation creates an auto-GVHD, a concept not mentioned by Samstein et al. Specifically, as the number of TMB increases, tumor cells with newly expressed neoantigens are no longer recognized as “self” and transformed into targets for patient’s own immune system cells. In other words, newly expressed neoantigens in malignant cells resulted in abrogation of the unresponsiveness/tolerance that existed between patient’s immune system and cancer cells, thus allowing for development of auto-GVHD with secondary therapeutic benefits, in analogy with GVM effects following allogeneic stem cell transplantation [2]. While a limited transformation is too weak in itself to provoke an effective T cell attack, the immune checkpoint blockade unleashes T cells against “altered-self” and tumors resulting in better overall survival [18]. This is consistent with the findings of Berner et al. [19], in non-small cell lung cancer. They demonstrated that T cells recognize and target shared tumor and skin antigens during checkpoint inhibitor therapy resulting in autoimmune-mediated skin toxicity and tumor regression. Not unexpectedly, the highest correlation (*r* = 0.678) was found between the TMB and ROR for <rash> among all AE terms in the FAERS database (Supplementary Table S3).

## Electronic supplementary material

Below is the link to the electronic supplementary material.

Supplementary material 1 (PDF 92 kb)

## Abbreviations

AE: Adverse event
FAERS: Food and Drug Administration Adverse Event Reporting System
GVHD: Graft-versus-host disease
GVM: Graft-versus-malignancy
ICI: Immune checkpoint inhibitor
irAE: Immune-related adverse event
NSCLC: Non-small cell lung carcinoma
ROR: Reporting odds ratio
TMB: Tumor mutation burden
