# Supplementary material for: Significant association between tumor mutational burden and immune-related adverse events during immune checkpoint inhibition therapies
Source: Cancer Immunol Immunother. 2020 Mar 9;69(5):683–7. doi: 10.1007/s00262-020-02543-6 (PMC7183506; doi:10.1007/s00262-020-02543-6)
Supplement: Supplementary file 1 — Supplementary material 1 (PDF 92 kb) [file 262_2020_2543_MOESM1_ESM.pdf]

Table S1. Search Terms of Immune-Related Adverse Events

rash	dermatitis bullous	enterocolitis	tubulointerstitial nephritis	episcleritis
pruritus	autoimmune dermatitis	autoimmune colitis	nephritis	scleritis
vitiligo	dermatitis exfoliative	colitis ulcerative	myocarditis	vogt-koyanagi-harada syndrome
pemphigoid	dermatitis exfoliative generalised	colitis ischaemic	autoimmune myocarditis	conjunctivitis
leukoderma	dermatitis acneiform	enterocolitis haemorrhagic	pericarditis	type 1 diabetes mellitus
psoriasis	autoimmune hepatitis	colitis microscopic	pericarditis malignant	fulminant type 1 diabetes mellitus
rash erythematous	hepatitis	autoimmune haemolytic anaemia	autoimmune pericarditis	autoimmune pancreatitis
rash generalised	immune-mediated hepatitis	immune thrombocytopenic purpura	myositis	pancreatitis
rash macular	hepatitis acute	histiocytosis haematophagic	polymyositis	pancreatitis acute
rash maculo-papular	hepatitis fulminant	sjogren's syndrome	arthritis	myasthenia gravis
rash morbilliform	autoimmune hypothyroidism	sialoadenitis	rheumatoid arthritis	encephalitis autoimmune
rash papular	autoimmune thyroiditis	polymyalgia rheumatica	polyarthritis	encephalitis
rash pruritic	thyroiditis	cytokine release syndrome	synovitis	myelitis
rash pustular	autoimmune thyroid disorder	autoimmune nephritis	peripheral arthritis	myelitis transverse
rash vesicular	hypophysitis	autoimmune endocrine disorder	arthritis reactive	arachnoiditis
pruritus generalised	lymphocytic hypophysitis	latent autoimmune diabetes in adults	seronegative arthritis	paraneoplastic encephalomyelitis
stevens-johnson syndrome	pneumonitis	autoimmune neuropathy	autoimmune arthritis	meningitis aseptic
erythema multiforme	radiation pneumonitis	autoimmune anaemia	uveitis	guillain-barre syndrome
toxic epidermal necrolysis	acute interstitial pneumonitis	autoimmune pancytopenia	autoimmune uveitis	encephalomyelitis
dermatitis	autoimmune lung disease	immune-mediated adverse reaction	iridocyclitis	limbic encephalitis
dermatitis psoriasiform	colitis	autoimmune disorder	iritis	noninfective encephalitis

**Table S2**					
**Organ**	**GVHD symptoms NIH (Jagasia et al 2015)**	**Associated terms in FAERS**			
	https://www.doi.org/10.1016/j.bbmt.2014.12.001	https://open.fda.gov/data/faers/			
	Distinctive symproms (Table 1) are red colored				
Skin:					
	Poikiloderma	Poikiloderma			
	Lichen planus-like features	Lichen planus			
	Sclerotic features				
	Morphea-like features				
	Lichen sclerosus-like features				
	Depigmentation	Skin depigmentation			
	Papulosquamous lesions	Rash papulosquamous			
	Sweat impairment	Sweat gland disorder			
	Ichthyosis	Ichthyosis			
	Keratosis pilaris	Keratosis pilaris			
	Hypopigmentation	Skin hypopigmentation			
	Hyperpigmentation	Skin hyperpigmentation			
	Erythema	Erythema			
	Maculopapular rash	Rash maculo-papular			
	Pruritus	Pruritus, Pruritus generalised			
Nails:					
	Dystrophy	Nail dystrophy			
	Longitudinal ridging, splitting or brittle features	Nail ridging			
	Onycholysis	Onycholysis			
	Pterygium unguis	Pterygium			
	Nail loss (usually symmetric, affects most nails)	Nail growth abnormal			
Scalp and Body Hair:					
	New onset of scarring or non-scarring scalp alopecia (after recovery from chemoradiotherapy)				
	Loss of body hair				
	Scaling				
	Thinning scalp hair, typically patchy, coarse or dull (not	Hair texture abnormal			
	Premature gray hair	Hair colour changes			
Mouth:					
	Lichen planus-like changes				
	Xerostomia	Dry mouth			
	Mucoceles				
	Mucosal atrophy	Mucosal atrophy			
	Ulcers	Mouth ulceration			
	Pseudomembranes				
	Gingivitis	Gingivitis			
	Mucositis				
	Erythema	Erythema			
	Pain	Oral pain			
Eyes:					
	New onset dry, gritty, or painful eyes	Eye pain,Dry eye			
	Photophobia	Photophobia			
	Periorbital hyperpigmentation				
	Cicatricial conjunctivitis				
	Keratoconjunctivitis sicca				
	Confluent areas of punctate keratopathy				
	Blepharitis (erythema of the eye lids with edema)	Blepharitis			
Genitalia:					
	Lichen planus-like features				
	Lichen sclerosus-like features				
	Erosions				
	Fissures				
	Ulcers				
	Females: Vaginal scarring or clitoral/labial agglutination				
	Males: Phimosis or urethral/meatus scarring or stenosis				
GI Tract:					
	Esophageal web	Acquired oesophageal web			
	Strictures or stenosis in the upper to mid third of the esophagus				
	Exocrine pancreatic insufficiency	Pancreatic insufficiency			
	Anorexia	Anorexia nervosa			
	Nausea	Nausea			
	Vomiting	Vomiting			
	Diarrhea	Diarrhoea			
	Weight loss	Weight decreased,Abnormal loss of weight			
	Failure to thrive (infants and children)	Failure to thrive			
Liver:					
	Total bilirubin, alkaline phosphatase > 2 × upper limit of	Liver function test abnormal			
	ALT> 2× upper limit of normal				
Lung:					
	Bronchiolitis obliterans diagnosed with lung biopsy	Obliterative bronchiolitis			
	Bronchiolitis obliterans syndrome (BOS)				
	Air trapping and bronchiectasis on chest CT				
	Cryptogenic organizing pneumonia (COP)				
	Restrictive lung disease	Restrictive pulmonary disease			
Muscles,Fascia,Joints:					
	Fasciitis	Fasciitis			
	Joint stiffness or contractures secondary to fasciitis or sclerosis				
	Myositis or polymyositis	Myositis,Polymyositis			
	Edema	Oedema peripheral,Oedema,Angioedema			
	Muscle cramps				
	Arthralgia or arthritis	Arthralgia,Rheumatoid arthritis,Arthritis			
Hematopoieticand Immune:					
	Thrombocytopenia	Thrombocytopenia			
	Eosinophilia	Eosinophilia			
	Lymphopenia	Lymphopenia			
	Hypo- or hyper-gammaglobulinemia	Blood immunoglobulin G decreased, Blood immunoglobulin G increased			
	Autoantibodies (AIHA, ITP)				
	Raynaud's phenomenon	Raynaud's phenomenon			
Other:					
	Pericardial or pleural effusions	Pericardial effusion			
	Ascites	Ascites			
	Peripheral neuropathy	Neuropathy peripheral			
	Nephrotic syndrome	Nephrotic syndrome			
	Myasthenia gravis	Myasthenia gravis			
	Cardiac conduction abnormality or cardiomyopathy	Cardiomyopathy			

Table S3

AE term (FAERS)	No. cases during ICI therapy	ROR vs TMB correlation (r)	Is irAE?	Is cGVHD-related AE?
Rash	970	0.678	irAE	
Drug ineffective	263	0.647		
Arthralgia	492	0.616		cGVHD-related AE
Hyperthyroidism	307	0.602		
Autoimmune colitis	171	0.600	irAE	
Hypothyroidism	641	0.547		
Prescribed overdose	249	0.546		
Pruritus	544	0.529	irAE	cGVHD-related AE
Immune-mediated adverse reaction	173	0.489	irAE	
Colitis	1170	0.481	irAE	
Hepatic enzyme increased	162	0.480		
Hyponatraemia	358	0.449		
Erythema	146	0.449		cGVHD-related AE
Malaise	467	0.442		
Enterocolitis	166	0.441	irAE	
Pain in extremity	164	0.439		
Diabetes mellitus	192	0.435		
Insomnia	173	0.407		
Hypokalaemia	166	0.407		
Hepatic function abnormal	359	0.406		
Type 1 diabetes mellitus	195	0.405	irAE	
Hypophysitis	603	0.389	irAE	
Thyroiditis	147	0.389	irAE	
Metastases to central nervous system	179	0.385		
Pneumonia	703	0.380		
Pyrexia	1387	0.377		
Myalgia	239	0.367		
Neuropathy peripheral	177	0.363		cGVHD-related AE
Respiratory failure	301	0.358		
Thrombocytopenia	298	0.328		cGVHD-related AE
Alanine aminotransferase increased	313	0.317		
Infection	167	0.307		
Interstitial lung disease	559	0.284		
Dizziness	248	0.281		
Liver disorder	232	0.278		
Hypopituitarism	149	0.266		
Lung disorder	200	0.260		
Diabetic ketoacidosis	185	0.255		
Neutropenia	167	0.252		
Arthritis	190	0.251	irAE	cGVHD-related AE
Diarrhoea	1682	0.249		cGVHD-related AE
Decreased appetite	724	0.233		
Weight decreased	466	0.225		cGVHD-related AE
Infusion related reaction	223	0.225		
General physical health deterioration	439	0.212		
Platelet count decreased	210	0.204		
Cough	363	0.199		
Hepatitis	312	0.198	irAE	
Autoimmune hepatitis	253	0.183	irAE	
Renal impairment	171	0.175		
Pancreatitis	170	0.159	irAE	
Muscular weakness	274	0.155		
Headache	548	0.153		
Myasthenia gravis	144	0.147	irAE	cGVHD-related AE
Constipation	314	0.128		
Back pain	314	0.110		
Chest pain	155	0.103		
Malignant neoplasm progression	4569	0.083		
Febrile neutropenia	146	0.082		
Fatigue	1275	0.077		
Pneumonitis	679	0.077	irAE	
Aspartate aminotransferase increased	307	0.071		
Pleural effusion	384	0.069		
Blood alkaline phosphatase increased	151	0.068		
Anaemia	522	0.065		
Dyspnoea	932	0.048		
Urinary tract infection	224	0.043		
Adrenal insufficiency	397	0.032		
Pulmonary embolism	245	0.019		
Chills	221	0.010		
Dysphagia	169	0.005		
Fall	198	0.001		
Renal failure	262	-0.011		
Myositis	213	-0.011	irAE	cGVHD-related AE
Sepsis	407	-0.019		
Cardiac failure	161	-0.020		
Myocarditis	239	-0.021	irAE	
Nausea	854	-0.034		cGVHD-related AE
Atrial fibrillation	226	-0.038		
Dehydration	441	-0.065		
Blood creatinine increased	186	-0.094		
Asthenia	539	-0.096		
Gait disturbance	139	-0.117		
Seizure	192	-0.128		
Oedema peripheral	163	-0.136		cGVHD-related AE
Abdominal pain	402	-0.148		
Disease progression	163	-0.149		
Acute kidney injury	504	-0.162		
Pain	414	-0.172		
Hyperglycaemia	203	-0.173		
Hypertension	169	-0.210		
Hypotension	277	-0.259		
Vomiting	667	-0.285		cGVHD-related AE
Confusional state	230	-0.287		
Adverse event	568	-0.367		
Ascites	152	-0.427		cGVHD-related AE
Product use in unapproved indication	1774	-0.451		
Off label use	889	-0.457		
Death	3042	-0.544		
Product use issue	989	-0.610		
